# Draft Genome Sequence of Lactobacillus paraplantarum OSY-TC318, a Producer of the Novel Lantibiotic Paraplantaracin TC318

**DOI:** 10.1128/MRA.00274-19

**Published:** 2019-05-09

**Authors:** Walaa E. Hussein, En Huang, Ismet Ozturk, Ahmed E. Yousef

**Affiliations:** a Department of Food Science and Technology, The Ohio State University, Columbus, Ohio, USA; b Department of Environmental and Occupational Health, University of Arkansas for Medical Sciences, Little Rock, Arkansas, USA; c Pro-Tek Analytical and Industrial Systems—3M Food Safety Department, Istanbul, Turkey; d Department of Microbiology, The Ohio State University, Columbus, Ohio, USA; University of Arizona

## Abstract

Lactobacillus paraplantarum OSY-TC318 was isolated from Turkish Tulum cheese and found to produce a potent anti-Gram-positive peptide. Sequencing of the OSY-TC318 genome revealed a genome size of 3,587,488 bp and an average GC content of 43.4%. Mining of the OSY-TC318 draft genome sequence revealed the gene cluster responsible for the biosynthesis of paraplantaricin TC318.

## Abstract (precis)

Lactobacillus paraplantarum OSY-TC318 was isolated from Turkish Tulum cheese and found to produce a potent anti-Gram-positive peptide. Sequencing of the OSY-TC318 genome revealed a genome size of 3,587,488 bp and an average GC content of 43.4%.

## ANNOUNCEMENT

When used to manufacture fermented foods, lactic acid bacteria (LAB) contribute not only to a product’s flavor and texture but also to its safety (1, 2). Few LAB strains produce lantibiotics (3). Recently, Lactobacillus paraplantarum OSY-TC318 was isolated from the Turkish cheese Tulum. The strain produces a lantibiotic (designated paraplantaracin TC318) which inhibits Gram-positive bacteria. Antimicrobial activity was determined as described previously (4). To better understand the biosynthetic pathway of paraplantaricin TC318, the draft genome sequence of *L. paraplantarum* OSY-TC318 was determined.

A single colony of strain OSY-TC318 was cultivated in de Man-Rogosa-Sharpe (MRS) broth (Oxoid, Thermo Scientific) at 30°C for 24 hours, and its genomic DNA (gDNA) was extracted and purified using a DNeasy blood and tissue kit (catalog number 69504; Qiagen, Valencia, CA). The analytical material source was Thermo Fisher Scientific, MA, unless indicated otherwise. The concentration of extracted gDNA was determined using a NanoDrop ND-1000 spectrophotometer. The DNA library was prepared using the Ion AmpliSeq library kit 2.0 (catalog number 4480441) and indexed with an Ion Xpress barcode adapters 1-16 kit (catalog number 4471250). Final library quality control was performed using an Ion Sphere quality control kit (catalog number 4468656). The library’s DNA concentration was confirmed by quantitative PCR (qPCR) using an Ion library TaqMan quantitation kit (catalog number 4468802). The library was analyzed using a 2100 Bioanalyzer (Agilent Technologies) to confirm its size. The sequencing was performed using Ion Torrent next-generation sequencing technology (Life Technologies, San Francisco, CA).

Sequencing results were analyzed using various software programs at their default settings, unless otherwise specified. Torrent Suite 4.4.2 was used to generate raw sequence reads after trimming low-quality reads. The raw data, 1,499,819 short sequencing reads (quality score, ≥15), were *de novo* assembled using CLC Genomics Workbench 9.0 (Qiagen) with the parameters mapping mode; minimum contig length, 200; automatic word size, 21; and automatic bubble size, 161. The assembly generated 412 contigs with an *N*_50_ value of 34,888 bp, a maximum contig size of 215,317 bp, a draft genome size of 3,587,488 bp, and an average GC content of 43.4%. Contig reordering was done against the reference genome of *L. paraplantarum* DSM 10667, which has the highest identity to OSY-TC318, as determined by BLASTN using progressiveMauve 2.4.0 (5).

The average nucleotide identity (ANI) values between OSY-TC318 and three sequenced *L. paraplantarum* genomes were calculated using the JSpeciesWS Web server (6). OSY-TC318 shared the highest ANI with *L. paraplantarum* DSM 10667 (97.82%), followed by *L. paraplantarum* AS-7 (97.75%) and *L. paraplantarum* L-ZS9 (96.81%). Annotation of the draft genome using the Rapid Annotations using Subsystems Technology (RAST) server (7) revealed 4,058 protein-coding sequences. The OSY-TC318 chromosome also contains 57 tRNA genes and 1 transfer-messenger RNA (tmRNA) gene, as predicted by the software ARAGORN (8). antiSMASH 4.1.0, (9) BAGEL3 (10), and NCBI-BLASTP (11) were used to identify the paraplantaracin biosynthetic gene cluster in the OSY-TC318 draft genome.

### Data availability.

The *L. paraplantarum* OSY-TC318 genome was deposited in the NCBI database under the BioProject accession number PRJNA517511. Raw reads were deposited in the Sequence Read Archive (SRA) under accession number SRR8526927. The assembled genome sequence was deposited in DDBJ/ENA/GenBank under accession number SEHH00000000. The version described in this paper is version SEHH01000000.
